# LncRNA GABPB1-AS1 and GABPB1 regulate oxidative stress during erastin-induced ferroptosis in HepG2 hepatocellular carcinoma cells

**DOI:** 10.1038/s41598-019-52837-8

**Published:** 2019-11-07

**Authors:** Wenchuan Qi, Zhenhua Li, Longjiang Xia, Jiangshan Dai, Qiao Zhang, Chuanfang Wu, Si Xu

**Affiliations:** 1 Key Laboratory of Bio-Resource and Eco-Environment of Ministry of Education, College of Life Sciences, Sichuan University, 610065 Chengdu, Sichuan P.R. China; 2 Affiliated Hospital of Changchun University of Traditional Chinese Medicine, 130117 Changchun, Jilin P.R. China; 3 Chengdu University of Traditional Chinese Medicine, 611130 Chengdu, Sichuan P.R. China; 4 China-Japan Union Hospital of Jilin University, 130033 Changchun, Jilin P.R. China; 5 Sichuan Academy of Medical Sciences and Sichuan Provincial People’s Hospital, 610072 Chengdu, Sichuan P.R. China

**Keywords:** Oncogenes, Cell death

## Abstract

Ferroptosis is a non-apoptotic, iron-dependent oxidative form of cell death that is specifically induced by erastin in RAS mutant cancer cells. Ferroptotic cell death is the result of membrane lipid peroxide damage caused by the accumulation of hydroxyl radicals derived from H_2_O_2_ by the Fenton reaction. Peroxidases are key cellular antioxidant enzymes that block such damaging processes. Few studies have examined the roles of long non-coding RNAs (lncRNAs) in the regulation of cellular oxidative stress, especially in ferroptosis. Here, we demonstrated that erastin upregulated the lncRNA GABPB1-AS1, which downregulated GABPB1 protein levels by blocking GABPB1 translation, leading to the downregulation of the gene encoding Peroxiredoxin-5 (PRDX5) peroxidase and the eventual suppression of the cellular antioxidant capacity. Such effects critically inhibited the cellular antioxidant capacity and cell viability. Additionally, high expression levels of GABPB1 were correlated with poor prognosis of hepatocellular carcinoma (HCC) Patients, while high GABPB1-AS1 levels in HCC patients correlated with improved overall survival. Collectively, these data demonstrate a mechanistic link between GABPB1 and its antisense lncRNA GABPB1-AS1 in erastin-induced ferroptosis and establish GABPB1 and GABPB1-AS1 as attractive therapeutic targets for HCC.

## Introduction

Ferroptosis is an oxidative form of cell death that is related to cancer suppression and neurodegenerative diseases^1–3^. Its iron dependence suggests that the inactivation of peroxidase may be a very important event in ferroptosis. Peroxidase blocks the production of hydroxyl radicals from H_2_O_2_ in the presence of iron ions and then prevents the accumulation of lipid reactive oxygen species (ROS)^2^. Previous studies have reported that erastin inhibits system xc- and triggers ferroptosis by blocking the antioxidant defence from obtaining reducing equivalents from glutathione (GSH), which leads to the accumulation of lipid ROS^4,5^. However, as the peroxidase system can obtain reducing equivalents from both GSH and NAD(P)H, even after depleting GSH, the peroxidase system can still maintain certain antioxidative stress ability with the help of NAD(P)H^6,7^. It is possible that there is an unknown mechanism of erastin in regulating the antioxidant capacity of the cell and demonstrating whether this mechanism is related to the ferroptosis process will help expand our understanding of the mechanism and role of ferroptosis in cancer suppression. Long noncoding RNAs (lncRNAs) represent a class of important molecules that play regulatory roles in multiple physiological and pathological processes in the cell^8–10^. However, a role for lncRNAs in the regulation of oxidative stress has not been clearly demonstrated.

Peroxiredoxins (PRDXs) are a family of non-selenium-dependent glutathione peroxidases that reduce peroxides, organic hydroperoxides and peroxynitrite^11^. Cells use PRDX pathways as enzymatic antioxidant defence systems to prevent oxidative damage caused by ROS^12^. The PRDX gene family includes six isoforms in mammals: Typical 2-cysteine PRDXs (PRDXs 1–4), atypical 2-cysteine PRDX (PRDX5), and atypical 1-cysteine PRDX (PRDX6)^13^. PRDX1 is mainly located in the cytoplasm, and knockdown of PRDX1 in mice leads to haemolytic anaemia and a shortened life span^14^. PRDX3 is located in the mitochondria, protects mitochondria against oxidative damage and affects diverse cellular processes, including growth, differentiation, carcinogenesis and apoptosis^15^. PRDX6 was identified as a secretory antioxidant protein of the olfactory epithelium^16^. A new study has revealed that PRDX6 plays an essential role in protecting cells against ferroptosis^17^. PRDX5 is localized widely in cells, including in the cytosol, mitochondria, peroxisome and nucleus and is able to reduce hydrogen peroxide, alkyl hydroperoxides, and peroxynitrite^18^. Although PRDX5 is involved in redox regulation in various metabolic functions, it remains unknown whether the function of this protein is related to ferroptosis.

Nuclear respiration factor-2 (NRF2), also called GA-binding protein, is a transcription activator^19^ that not only regulates the expression of genes encoding mitochondrial respiratory chain proteins and mitochondrial transport proteins^20–22^ but also affects the cellular antioxidant capacity by regulating the expression of genes encoding peroxidases, such as PRDX5. NRF2 has three subunits, alpha, beta and gamma, and GABPB1 protein is the activation subunit of NRF2^23^. GABPB1 forms a tetrameric complex with the alpha subunit and stimulates the transcription of various genes^24,25^ including the antioxidant gene PRDX5^26^. PRDX5 is expressed at a high level in a variety of mammalian cell lines and normal tissues^27^. The transcription factor GABP/NRF-2 is required for the basal expression of the human PRDX5 gene, and it was also found that mutation-mediated inactivation of all potential GAPB binding sites in the PRDX5 promoter led to 80% inhibition of its basal activity in a reporter gene assay^26^. The GABPB1-AS1 lncRNA is the antisense RNA of GABPB1 mRNA. The expression level of GABPB1-AS1 changes in response to various stimuli such as stress^28,29^ but the physiological significance and molecular mechanism are not clear.

In this study, we analysed the regulatory effects of lncRNA GABPB1-AS1 on GABPB1 and its potential functions in HepG2 hepatocellular carcinoma cell ferroptosis induced by erastin. We found that GABPB1-AS1 was upregulated by erastin which inhibited the translation of GABPB1, leading to the inhibition of peroxidase gene expression and causing the accumulation of ROS and MDA and HepG2 cell death. These findings revealed that GABPB1-AS1 may be a key molecule in HCC cell ferroptosis induced by erastin and enrich our understanding of lncRNA regulation of oxidative stress.

## Materials and Methods

### Cell line and treatments

HepG2, Huh7, and Hep3B cells were purchased from the Cell Bank of the Chinese Academy of Sciences (Shanghai, China). Cells were cultured in DMEM (HyClone, Logan, UT, USA) containing 100 U/ml penicillin, 100 µg/ml streptomycin and 10% foetal bovine serum (Gibco, Carlsbad, CA, USA) at 37 °C in 5% CO_2_. Erastin and ferrostatin-1 were obtained from MedChemExpress (Monmouth Junction, NJ, USA).

### RNA extraction and real-time quantitative PCR (RT-qPCR)

RNA was extracted using TRIzol reagent (Thermo Scientific, Waltham, MA, USA), treated with DNase I (Thermo Scientific) and converted into cDNA using random hexamer primers and RevertAid Reverse Transcriptase (Thermo Scientific). RT-qPCR was performed using SYBR Premix Ex Taq II (TaKaRa, Dalian, China) and a StepOnePlus Real-Time PCR System (Applied Biosystems, Thermo Scientific). Data were analysed using the relative quantification (2^−ΔΔCT^) method. *GAPDH* mRNA was used as a reference gene for mRNA quantification. The primer sequences for PCR are listed in Supplementary Table 1.

### Western blot

Western blotting was performed according to standard methods. Cell lysates were extracted using NP-40 lysis buffer (50 mM Tris-HCl, pH 7.4, 150 mM NaCl, 1% NP-40, 0.5% NaDC, 0.1% SDS), and protein concentrations in the cell lysates were measured using the BCA Protein Assay kit (Thermo Scientific). Equal amounts of proteins were separated by sodium dodecyl sulfate-polyacrylamide gel electrophoresis and then transferred to nitrocellulose membranes. After incubation with PBS with 0.2% Tween 20 (PBST) containing 5% skimmed milk powder (SMP) for 1 h, the membranes were incubated overnight at 4 °C with primary antibodies diluted at 1:500–1:5000 in PBST containing 5% SMP. After washing five times with PBST, the membranes were incubated with secondary antibodies diluted at 1:10,000 in PBST containing 5% SMP at room temperature for 1 h. After washing five times again with PBST, the bound antibodies were visualized using ECL chemiluminescence (Thermo Scientific) on X-ray films (Carestream Health, Shanghai, China). Actin was used as a loading control. Antibodies against GABPB1 (1:1000), PRDX5 (1:1000), actin (1:5000), eIF4A (1:1000), Flag (1:2000) and horseradish peroxidase-conjugated secondary antibodies were purchased from ProteinTech (Chicago, IL, USA). Antibodies against caspase-3 (1:1000) and LC3 I/II (1:1000) were purchased from Cell Signaling Technology (Danvers, MA, USA).

### Vector construction, small interfering RNA (siRNA) synthesis and cell transfection

The cDNA encoding the coding sequence of GABPB1 was PCR-amplified using PrimeStar Max PCR mix(Takara) and subcloned into the NheI and BamHI (Thermo Scientific) sites of the pcDNA3.1 vector. For the expression of GABPB1-AS1 RNA, the cDNA sequence was PCR-amplified using PrimeStar Max PCR mix and subcloned into the NheI and BamHI sites of the pcDNA3.1 vector. The cDNA encoding PRDX5 amplified by PCR was amplified using PrimeStar PCR mix and subcloned into the EcoRI and BamHI sites of the pcDNA3.1 vector. The siRNAs targeting GABPB1, GABPB1-AS1 and the control siRNA were synthesized by Genepharm (Shanghai, China). Transfection of plasmids and siRNAs was conducted using Lipofectamine 3000 (Thermo Scientific) according to the manufacturer’s protocol. The sequences of the cloning primers and siRNAs are listed in Supplementary Table 1.

### Chromatin immunoprecipitation (ChIP)

ChIP was performed on the chromatin extract from HepG2 cells according to the instructions provided by Merck-Millipore. ChIP-derived DNA was quantified using real-time qPCR analysis. The primers used are shown in Supplementary Table 1.

### RNA pull-down

The GABPB1 first exon (1–424 nucleotides, sense direction) and GABPB1 first exon (1–424 nucleotides, antisense direction) were amplified with primers and biotin-labelled with the Biotin RNA Labeling Mix (Roche, Basel, Switzerland) by *in vitro* transcription. The *in vitro*-transcribed transcripts were treated with RNase-free DNase I (Roche) and purified with TRIzol reagent. Cell lysates from 1 × 10^6^ cells were combined with 1 mL IP buffer (25 mM Tris-Cl [pH 7.4], 150 mM NaCl, 0.5% NP-40, 0.5 mM DTT and 1 × complete protease inhibitors [Roche]) supplemented with 100 U/ml RNase Inhibitor (Thermo Scientific). HepG2 cell lysate was incubated with 3 μg of purified biotinylated transcripts for 1 h at 25 °C, and then complexes were isolated with streptavidin-coupled T1 beads (Dynabeads, Thermo Scientific). The RNA present in the pulldown material was examined by RT-PCR. The primers used are shown in Supplementary Table 1.

### Isolation of cytoplasmic and nuclear RNA

Cytoplasmic and nuclear proteins were separated using the Nuclear and Cytoplasmic Protein Extraction Kit (Beyotime, Shanghai, China) according to the manufacturer’s instructions. RiboLock RNase Inhibitor 100 U/ml (Thermo Scientific) was added to the lysis buffer from the kit. The nuclear precipitate was directly resuspended in TRIzol reagent and subjected to RNA extraction.

### RNase protection assay (RPA)

RNA was isolated from HepG2 cells and incubated at 37 °C for 1 h and then treated with RNase A + T (1 U/ml, Thermo Scientific) for 30 min at 37 °C. RNase A + T digests single-stranded RNA but not RNA duplexes. After RNase A + T treatment, the RNA was extracted using TRIzol. cDNA was generated, and real-time qPCR was performed as described above. The primers used are shown in Supplementary Table 1.

### Polysome analysis

Polysome analysis was performed as previously described^30^. At least 50 million HepG2 cells were treated with cycloheximide (CHX) (0.1 mg/ml) (Solarbio, Shanghai, China) for 15 min. Cells were collected and washed with ice-cold PBS containing CHX (0.1 mg/ml) three times, followed by centrifugation at 600 g for 5 min at 4 °C. Cells were then lysed in 1 ml of polysome extraction buffer (15 mM Tris-Cl [pH 7.4], 15 mM MgCl_2_, 0.3 M NaCl, 0.1 mg/ml CHX, 1 mg/ml heparin and 1% Triton X-100) and then centrifuged at 10,000 × g for 20 min at 4 °C. The supernatant was loaded onto a sucrose gradient of 10–50% (w/v) in an ultra-centrifuge (Beckman Coulter) at 100,000 g for 4 h. Absorbance at 254 nm was measured. RNA was isolated from the fractions by phenol-CHCl_3_ extraction and ethanol precipitation.

### RNA immunoprecipitation (RIP)

HepG2 cell lysate was prepared as described in the RNA pull-down assay section. Cell lysates from 1 × 10^6^ cells were immunoprecipitated using 2 μg eIF4A antibody or IgG at 4 °C overnight with rotation. The immunoprecipitates were digested with proteinase K (Thermo Scientific), and then the immunoprecipitated RNA was purified by TRIzol regent (Thermo Scientific). cDNA was synthesized with random primers using the RevertAid RT Kit (Thermo Scientific) and subjected to RT-qPCR for GABPB1 transcripts.

### Cell viability assay

Cell viability was measured using the CCK-8 kit (KeyGEN BioTECH, Nanjing, China) according to the manufacturer’s protocol. Briefly, HepG2 cells were seeded in 96-well plates at 5000 cells/well in triplicate. After 2 h incubation with fresh media containing 10% (v/v) CCK-8 reagent, the absorbance at 450 nm of each well was measured on a microplate reader. The mean value of the wells with media alone was used as background.

### Determination of ROS levels

Cells were incubated with 10 mM 2ʹ,7ʹ-dichlorofluorescein diacetate (DCFH-DA) (KeyGEN BioTECH) for 20 min and washed with PBS three times. Cellular fluorescence changes were observed under a fluorescence microscope (Olympus, Tokyo). The fluorescence intensity of cells was measured using a NovoCyte Flow Cytometer (ACEA Biosciences, China).

### Lipid peroxidation assay

The concentration of MDA, one of the end products of lipid peroxidation, was assessed using a lipid peroxidation assay kit purchased from Solarbio (Beijing, China) according to the manufacturer’s instructions.

### Apoptosis assay

The apoptotic ratio was measured by Annexin V-FITC/propidium iodide (PI) double staining (KeyGEN BioTECH, Nanjing, China) according to the manufacturer’s protocol, followed by flow cytometry analysis (BD Pharmingen, San Diego, CA).

### Autophagy assay

The cells were stained with monodansylcadaverine (MDC; KeyGEN BioTECH) for 25 min at 37 °C and observed under a fluorescence microscope (Olympus). The fluorescence intensity was analysed by a fluorescence microplate reader (Varioskan Flash; Thermo Fisher Scientific, Waltham, MA).

### Tissue samples from HCC patients

A total of 12 human HCC tissue samples were obtained from the Affiliated Hospital of Changchun University of Traditional Chinese Medicine. The tissues were snap-frozen in liquid nitrogen immediately after dissection and stored in liquid nitrogen until further analysis by western blotting and RT-qPCR. Studies using human tissues were approved by the Human Research Ethics Committees of the Changchun University of Traditional Chinese Medicine, and all participants provided written informed consent. This study followed ICH-GCP guidelines, the Declaration of Helsinki, internationally recognized ethical standards, and relevant Chinese laws and regulations. All collected samples were eligible for experimental use.

### Statistical analyses

Data are expressed as the means ± SD. Significant differences in the means between two groups were determined using the two-tailed Student’s t-test. Differences were considered significant at *P < *0.05* and **P < *0.01*.

## Results

### Decreased GABPB1 expression levels during erastin-mediated ferroptosis

**Figure 1:**
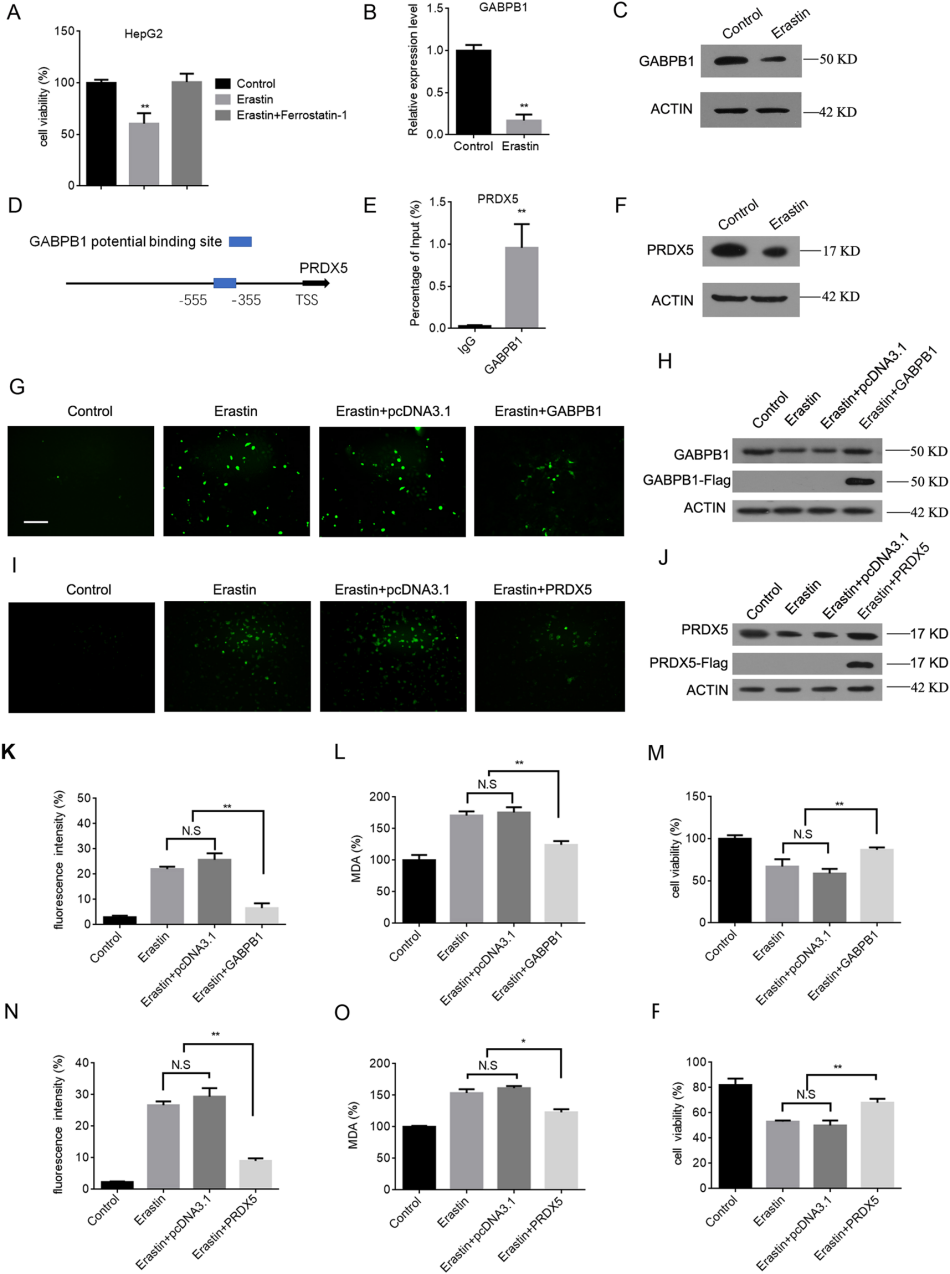
Decreased GABPB1 expression levels during ferroptosis in HepG2 cells. (**A**) HepG2 cells were treated with erastin (10 μM) or ferrostatin-1 (1 μM) as indicated for 24 h, and cell viability was determined using CCK-8 assays. (**B**,**C**) HepG2 cells were treated with erastin (10 μM) for 24 h, and GABPB1 mRNA (**B**) and protein (**C**) levels were determined. (**D**) Schematic illustration of the genomic locus of GABPB1 potential binding sites in the PRDX5 promoter. (**E**) ChIP and RT-qPCR assays confirmed the binding loci of GABPB1 to the PRDX5 promoter. (**F**) HepG2 cells were treated with erastin (10 μM) for 24 h, and PRDX5 protein levels were determined. (**G**,**I**) Untransfected HepG2 cells or cells transfected as indicated were treated with 10 μM erastin for 24 h. ROS generation was determined by DCFH-DA staining, and cells were observed under a fluorescence microscope. GABPB1 (**H**) and PRDX5 (**J**) protein levels were determined. Bars: 50 μm. (**K**,**N**) ROS were measured in cells treated as indicated by flow cytometry using DCFH-DA staining. (**L**,**O**) MDA levels were measured. (**M**,**P**) Cell viability was assayed in cells treated as indicated. Values are expressed as the means ± SD (n = 3). *P < 0.05, **P < 0.01.

Erastin is commonly used as an inducer of ferroptosis^31^, and previous studies have confirmed that 10 μM erastin induces an inhibitory effect on HCC cell growth^32^. CCK-8 assays showed that treatment of HepG2 cells with erastin for 24 h resulted in an ~40% reduction in HepG2 cell survival and that cell death was blocked by ferrostatin-1 (a potent ferroptosis inhibitor) (Fig. 1A). We next analysed the protein and mRNA levels of GABPB1 in HepG2 cells treated with erastin. Remarkably, erastin treatment decreased GABPB1 mRNA and protein expression in HepG2 cells (Fig. 1B,C). The human PRDX5 gene encodes a cytoprotective antioxidant enzyme that inhibits endogenous or exogenous peroxide accumulation and is regulated by the transcription factor GABP/NRF-2^33^. Bioinformatic analysis showed the potential GABP protein binding sites in the PRDX5 promoter^26^ (Fig. 1D). Chromatin immunoprecipitation (ChIP) and qPCR assays confirmed the binding loci of GABPB1 on the PRDX5 promotor (Fig. 1E). We also detected reduced protein levels of PRDX5 in HepG2 cells treated with erastin (Fig. 1F). To evaluate the role of GABPB1 in erastin-induced ROS and MDA generation, we overexpressed GABPB1 in HepG2 cells. Overexpression of GABPB1 reduced the ROS generation (Fig. 1G,K) and MDA levels induced by erastin (Fig. 1L) and restored cell viability in the presence of erastin (Fig. 1M). We further examined the role of the peroxidase protein PRDX5 in erastin-induced oxidative stress by overexpression of PRDX5 and observed reduced ROS generation and MDA levels and restored cell viability (Fig. 1I,N–P). To confirm that this observation was not cell line specific, we analysed the protein and mRNA levels of GABPB1 in two other HCC cell lines (Huh7 and Hep3B). The results showed that treatment with erastin significantly reduced GABPB1 expression in Huh7 and Hep3B cell lines (Supplementary Fig. 1a,b). Together, these results suggest that erastin may promote ROS generation and MDA levels by inhibiting the expression of GABPB1 and its target gene PRDX5.

### The GABPB1 antisense chain lncRNA GABPB1-AS1 is upregulated by erastin

**Figure 2:**
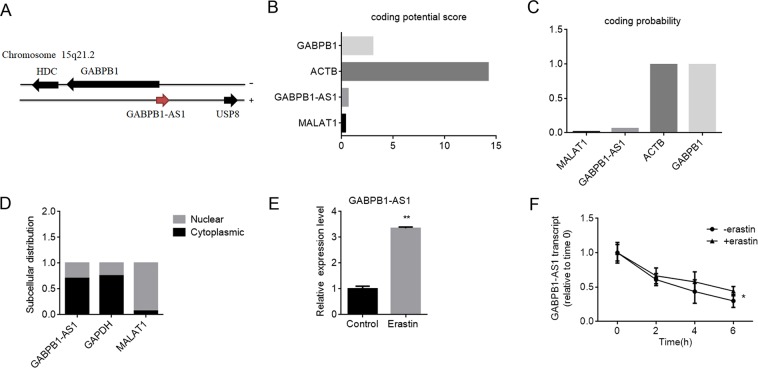
Increased GABPB1-AS1 expression levels during ferroptosis in HepG2 cells. (**A**) Schematic illustration of the genomic locus of GABPB1, GABPB1-AS1 and two neighbouring genes on human chromosome 15q21.2. The GABPB1 and HDC genes are encoded by the (−) DNA strand, while the GABPB1-AS1 and USP8 genes are encoded by the (+) DNA strand. (**B**,**C**) GABPB1-AS1 was predicted to be a poor protein coding capacity. The RNA sequences of GABPB1, ACTB, GABPB1-AS1 and MALAT1 were evaluated by the CPC (**B**) and CPAT (**C**) programmes. MALAT1 served as a control noncoding transcript, while GABPB1 and ACTB served as control protein coding transcripts. (**D**) Subcellular localization of GABPB1-AS1, GAPDH and MALAT1. GAPDH mRNA was used as a control for the cytoplasmic fraction; MALAT1 mRNA was used as a control for the nuclear fraction. (**E**) HepG2 cells were treated with erastin (10 μM) for 24 h, and GABPB1-AS1 mRNA levels were assayed. (**F**) The stability of GABPB1-AS1 mRNA over time was measured after blocking new RNA synthesis with α-amanitin (50 μM) and then treating the cells with (+) or without (−) erastin. GABPB1-AS1 mRNA levels were normalized to 18 S rRNA levels. Values are expressed as the means ± SD (n = 3). *P < 0.05, **P < 0.01.

The GABPB1 gene locus contains an antisense lncRNA named GABPB1-AS1 (gene ID: 100129387; location: 15q21.2), and the GABPB1 gene and GABPB1-AS1 are oriented in a “head to head” configuration (Fig. 2A). Prediction of the GABPB1-AS1 translation capacity using CPC (http://cpc.cbi.pku.edu.cn/)^34^ and CPAT (http://lilab.research.bcm.edu/cpat/index.php)^35^ showed that the GABPB1-AS1 protein-coding ability was poor (Fig. 2B,C). Nuclear separation experiments showed that the abundance of GABPB1-AS1 was much higher in the cytoplasmic fraction compared with the nuclear fraction (Fig. 2D). We also examined the effects of erastin on GABPB1-AS1 and found that erastin increased the expression of GABPB1-AS1 levels (Fig. 2E).

GABPB1-AS1 is a short-lived lncRNA. The expression levels of GABPB1-AS1 have been shown to be elevated because of prolonged decay rates in response to chemical stressors and interruption of RNA degradation pathways^29^. To determine whether the addition of erastin prolonged the half-life of GABPB1-AS1, HepG2 cells were treated with α-amanitin to block new RNA synthesis, and then the loss of GABPB1-AS1 mRNA was measured. The results showed that erastin prolonged the half-life of GABPB1-AS1 (Fig. 2F). Consistent with the findings in HepG2 cells, erastin also increased the expression levels of GABPB1-AS1 and prolonged the half-life of GABPB1-AS1 in both Huh7 and Hep3B cells (Supplementary Fig. 2a,b).

### GABPB1-AS1 regulates GABPB1 expression at the translational level

**Figure 3:**
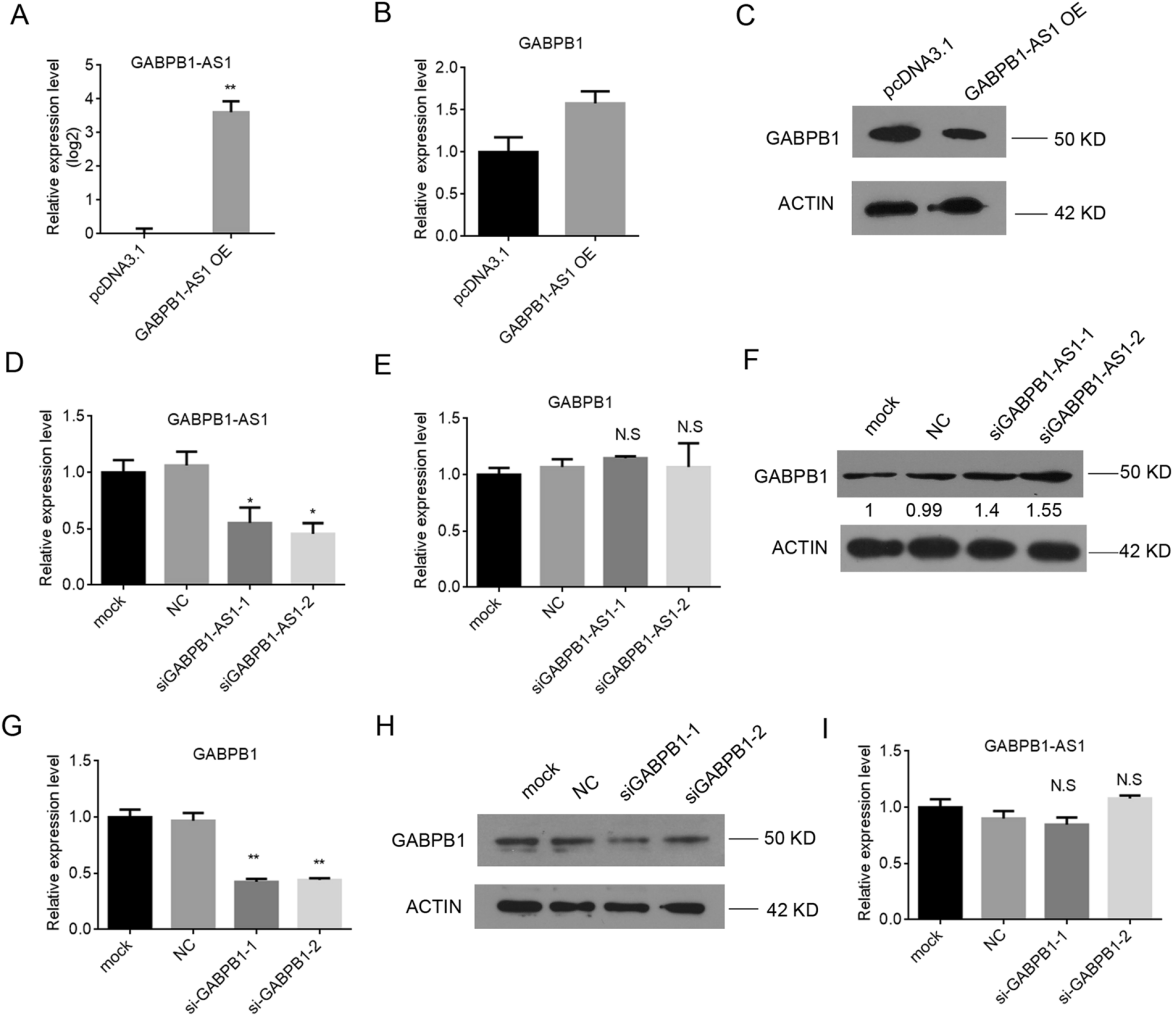
GABPB1-AS1 negatively regulates GABPB1 through a post-translational mechanism. (**A**) Relative expression of GABPB1-AS1 determined by RT-qPCR in HepG2 cells transfected with pcDNA3.1 or GABPB1-AS1 overexpression vector. (**B**) RT-qPCR results showing GABPB1 mRNA expression in HepG2 cells transfected with pcDNA3.1 or GABPB1-AS1 overexpression vector. (**C**) Western blot analysis of GABPB1 in HepG2 cells transfected with pcDNA3.1 or GABPB1-AS1 overexpression vector. ACTIN was used as a loading control for RNA and total protein levels. (**D**) RT-qPCR confirmed the downregulation of GABPB1-AS1 using two different siRNAs compared with the negative control (NC). (**E**,**F**) RT-qPCR and western blot analysis of GABPB1 mRNA levels (**E**) and protein levels (**F**) after GABPB1-AS1 expression was reduced. (**G**,**H**) RT-qPCR (**G**) and western blot (**H**) confirmed the downregulation of GABPB1 mRNA and protein by two different siRNAs. (**I**) RT-qPCR analysis of GABPB1-AS1 mRNA levels after GABPB1 expression was reduced. Values are expressed as the means ± SD (n = 3). *P < 0.05, **P < 0.01.

The above results showed that erastin decreased the expression of GABPB1 mRNA and protein while increasing the expression of GABPB1-AS1. These findings suggest that GABPB1-AS1 may have an inhibitory effect on the expression of GABPB1. We next constructed a GABPB1-AS1 expression vector for transfection into HepG2 cells to determine the effects of GABPB1-AS1 on GABPB1 mRNA and protein levels by RT-PCR and western blot analysis, respectively. We confirmed the strong upregulation of GABPB1-AS1 in HepG2 cells transfected with the GABPB1-AS1 vector (Fig. 3A). Overexpression of GABPB1-AS1 had a slight effect on the expression of GABPB1 mRNA (Fig. 3B). However, the protein level of GABPB1 was inhibited in cells overexpressing GABPB1-AS1 (Fig. 3C), along with decreased PRDX5 protein levels (Supplementary Fig. 3a).

We next designed siRNAs targeting GABPB1-AS1. While RT-qPCR results showed effective knockdown of GABPB1-AS1 using the siRNAs (Fig. 3D), no significant effect on the expression of GABPB1 mRNA was observed (Fig. 3E). However, western blot showed that GABPB1-AS1 knockdown resulted in increased GABPB1 protein levels (Fig. 3F). Since GABPB1-AS1-siRNA2 demonstrated the most efficient knockdown, this siRNA was used in subsequent experiments. To further elucidate the regulation of GABPB1 and GABPB1-AS1, we knocked down GABPB1 using two independent siRNAs. RT-qPCR results and western blot analysis showed effective knockdown of GABPB1 using the siRNAs (Fig. 3G,H). Knockdown of GABPB1 reduced PRDX5 protein levels (Supplementary Fig. 3b). However, there was no effect on the expression of GABPB1-AS1 (Fig. 3I). GABPB1-siRNA1 demonstrated the most efficient knockdown, and this siRNA was used in subsequent experiments. These results indicate that GABPB1-AS1 regulates the expression of GABPB1 through a post-transcriptional mechanism.

We further investigated whether knockdown of GABPB1 lead to cell apoptosis and autophagy. Interestingly, results from Annexin V-FITC/PI double staining and activation of caspase-3 further verified the roles of GABPB1 in cell apoptosis (Supplementary Fig. 3c,d). It has been shown that PRDX5 suppresses p53-dependent apoptosis^36^, and p53 can also enhance ferroptosis by inhibiting the expression of SLC7A11 (solute carrier family 7 member 11)^37^. We hypothesized that GABPB1 may participate in apoptosis by regulating PRDX5 and p53; however, whether the involvement of GABPB1 in ferroptosis is related to p53 needs to be further proven. It has been previously reported that GABP binds to the promoter regions of BECN1-PIK3C3 complex genes and activates their transcriptional activities. Knockdown of GABP reduced BECN1-PIK3C3 complex transcripts and reduced autophagy^38^. To investigate the effect of GABPB1 knockdown on HepG2 autophagy, AVOs (acidic vesicular organelles) were visualized using MDC staining, and western blot analysis was performed of microtubule-associated proteins 1 A/1B light chain 3 (LC3) II protein, the lipidated form of LC3 that is associated with the autophagosomal membrane. However, the results showed that knockdown of GABPB1 caused neither the formation of acidic vesicular organelles nor a change in LC3 II protein levels (Supplementary Fig. 3e–g). Overall, knockdown of GABPB1 leads to cell apoptosis but not autophagy.

### GABPB1-AS1 interacts with GABPB1 and suppresses GABPB1 translation

**Figure 4:**
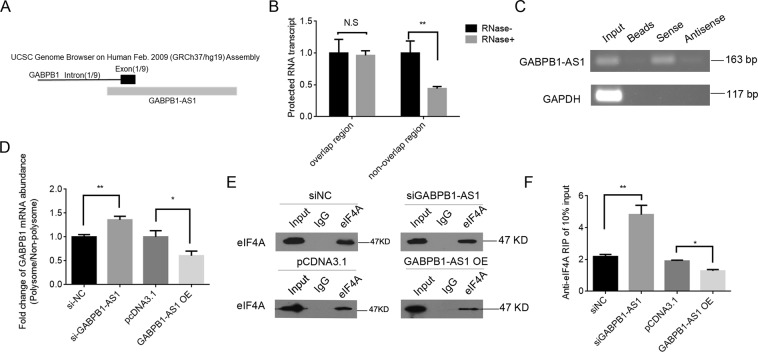
GABPB1-AS1 suppresses GABPB1 translation through direct binding with GABPB1 mRNA. (**A**) Schematic showing GABPB1-AS1 overlapping with the first exon of GABPB1. (**B**) RNase protection assay performed on RNA samples from HepG2 cells; GABPB1 mRNA regions overlapping and non-overlapping with GABPB1-AS1 were examined. (**C**) RNA pull-down assays were performed for endogenous GABPB1-AS1 mRNA using *in vitro* transcribed biotin-labelled GABPB1 exon 1 (sense) or antisense sequence. (**D**) Polysomes from HepG2 cells transfected with siGABPB1-AS1, GABPB1-AS1 overexpression vector or the respective controls were fractioned through sucrose gradients, and the relative distribution of GABPB1 mRNA in the gradients was measured by RT-qPCR. Data were normalized as polysome/nonpolysome. (**E**) Immunoprecipitation and western blot of eIF4A from HepG2 cells transfected with siGABPB1-AS1, GABPB1-AS1 overexpression vector or the respective controls. (**F**) RIP was performed for GABPB1 mRNA using anti-eIF4A in HepG2 cells transfected with siGABPB1-AS1, GABPB1-AS1 overexpression vector or the respective controls. Values are expressed as the means ± SD (n = 3). *P < 0.05, **P < 0.01.

Analysis of the GABPB1-AS1 and GABPB1 gene sequences revealed complementary fragments in the 812–1235 nt region of GABPB1-AS1 and the first exon (1–424 nt) of GABPB1 (Fig. 4A). To determine whether GABPB1 mRNA and GABPB1-AS1 overlap, we performed an RNase protection assay. The results showed protection of the GABPB1-AS1 and GABPB1 overlapping regions from RNase degradation and the formation of RNA duplexes (Fig. 4B). Furthermore, affinity pulldown of endogenous GABPB1-AS1 mRNA using *in vitro* transcribed biotin-labelled GABPB1 first exon showed that GABPB1 mRNA interacted with GABPB1-AS1 mRNA (Fig. 4C).

To further clarify how GABPB1-AS1 affects GABPB1 translation, polysomes were extracted from cells either depleted of GABPB1-AS1 or overexpressing GABPB1-AS1. RNA from the polysome and non-polysome fractions was extracted, and GABPB1 mRNA levels were evaluated by RT-qPCR. We found that the polysome-associated GABPB1 mRNA level was significantly increased in GABPB1-AS1 siRNA-transfected cells compared with controls, while the polysome-associated GABPB1 mRNA level was decreased in GABPB1-AS1-overexpressing cells compared with controls (Fig. 4D). These data indicated that GABPB1-AS1 suppressed GABPB1 translation by blocking the recruitment of GABPB1 mRNA to polysomes.

During translation, the eukaryotic initiation factor-4A (eIF4A) is required for the binding of mRNA to 40 S ribosomal subunits^39^. To further elucidate the functional mechanisms, we examined whether eIF4A binding with GABPB1 mRNA was inhibited in the regulation of GABPB1 translation by GABPB1-AS1. We performed anti-eIF4A RIP and found that the interaction between GABPB1 mRNA and eIF4A was significantly increased in cells depleted of GABPB1-AS1 and that GABPB1-AS1 overexpression reduced eIF4A binding to GABPB1 mRNA (Fig. 4E,F). Based on these results, we propose that GABPB1-AS1 suppresses GABPB1 translation by blocking GABPB1 mRNA recruitment to polysomes and binding with eIF4A.

### Abnormal expression of GABPB1-AS1 affects ROS and MDA levels in HepG2 cells and GABPB1 expression in clinical tissues

**Figure 5:**
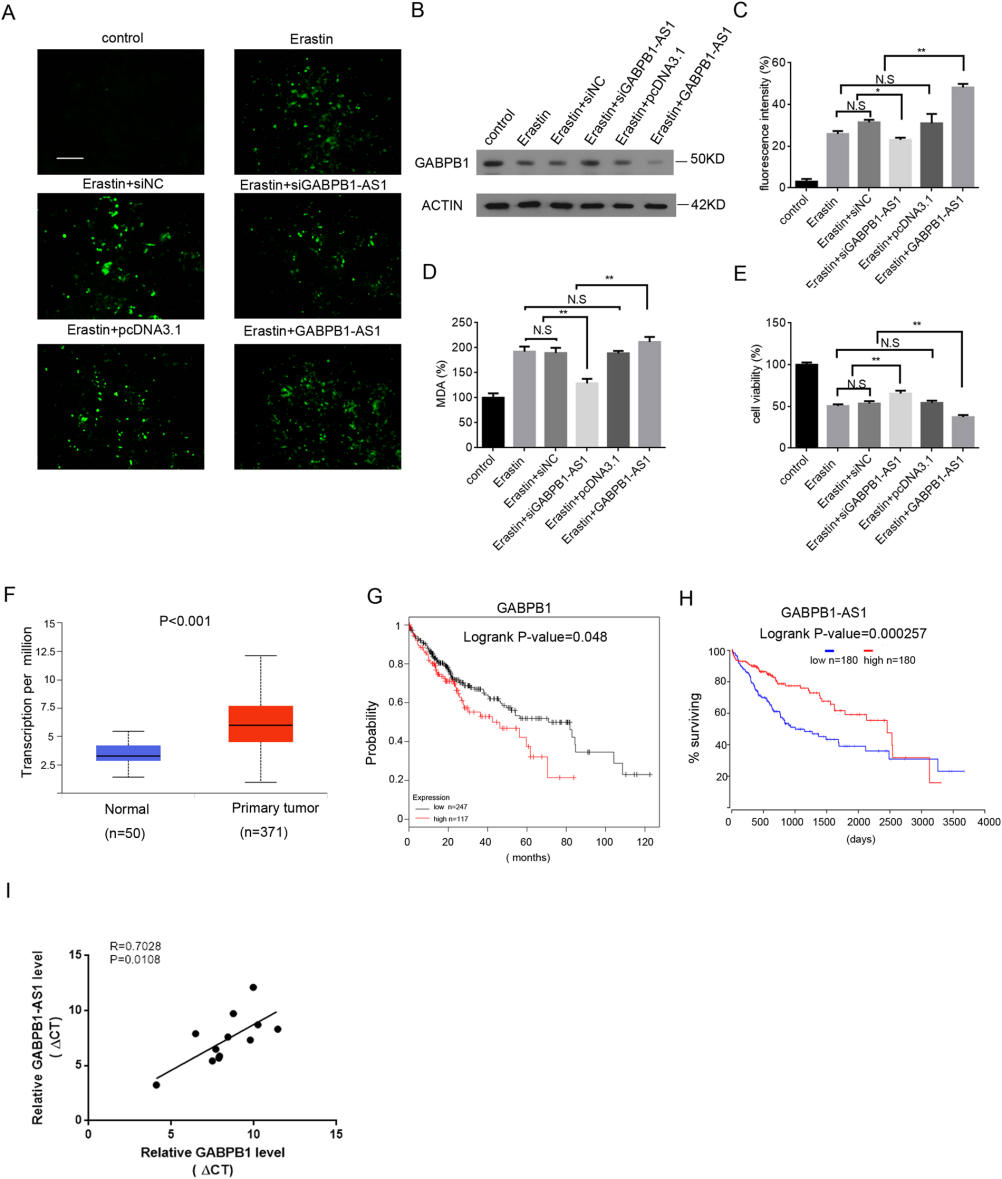
GABPB1-AS1 regulates the erastin-induced ROS and MDA levels and abnormal expression of GABPB1 in HCC tissues. (**A**) HepG2 cells were transfected with siGABPB1-AS1, GABPB1-AS1 overexpression vector or the respective controls and then treated with erastin (10 μM) for 24 h. DCFH-DA stained cells were observed by fluorescence microscopy. Bars: 50 μm. (**B**) Western blot analysis of GABPB1 protein in HepG2 cells treated as indicated. (**C**) ROS generation in cells treated as indicated was measured by flow cytometry using DCFH-DA staining. (**D**) MDA levels were measured in cells treated as indicated. **(E**) Cell viability was determined in cells treated as indicated by CCK-8 assays. (**F**) GABPB1 expression levels in normal (n = 50) and HCC primary tumour (n = 371) samples. (**G**) Kaplan-Meier analyses of the correlation between GABPB1 expression levels and overall survival. (**H**) Kaplan-Meier analyses of the correlation between GABPB1-AS1 expression levels and overall survival. (**I**) The expression levels of GABPB1-AS1 and GABPB1 were analysed using RT-qPCR in human HCC tissues (n = 12 independent samples), and the ΔCT (threshold cycle) values were normalized to GAPDH mRNA and subjected to Pearson correlation analysis. Values are expressed as the means ± SD (n = 3). *P < 0.05, **P < 0.01.

Ferroptosis is triggered by inactivation of cellular GSH-dependent antioxidant defences, leading to the accumulation of toxic lipid ROS^40^. We next examined the role of GABPB1-AS1 on ROS and MDA levels in cells stimulated by erastin. We found that GABPB1-AS1 knockdown could partially reduce the ROS and MDA levels of erastin-treated cells and could inhibit cell death. Overexpression of GABPB1-AS1 increased ROS and MDA levels as well as promoted cell death (Fig. 5A,C–E). Moreover, western blot analysis showed that GABPB1 protein was increased after GABPB1-AS1 knockdown in erastin-treated cells, and overexpression of GABPB1-AS1 inhibited the protein expression of GABPB1 (Fig. 5B). These results demonstrated that overexpression of GABPB1-AS1 may increase ROS generation, leading to membrane lipid peroxide damage, which increases cell death.

Cancer cells need higher antioxidant-producing capacity that enables them to survive oxidative stress^41,42^. We found that overexpression of GABPB1 could reduce ROS and MDA levels, which indicated that GABPB1 could enhance the cellular antioxidant capacity. We then investigated GABPB1 expression in human HCC tissues. Consistent with our assumption, the results showed higher GABPB1 expression in HCC tissues than in normal tissue^43^ (Fig. 5F). Low levels of ROS can activate various signalling pathways to stimulate survival and cell proliferation^44^. Kaplan-Meier analysis further demonstrated that high GABPB1 mRNA levels in HCC tissues correlated with reduced overall survival^45^. Excess levels of ROS irreversibly damage cellular macromolecular components and result in cancer cell death^46^. Our results showed that HCC cell death induced by high GABPB1-AS1 levels correlated with increased ROS and MDA levels. Kaplan-Meier analysis also revealed that high GABPB1-AS1 levels in HCC tissues correlated with improved overall survival^47^. Interestingly, RT-qPCR results showed a positive correlation between the levels of GABPB1-AS1 and GABPB1 transcripts in 12 human HCC tissues (Fig. 5H). This may be due to the overlap between GABPB1 mRNA and GABPB1-AS1, which could enhances the stability of GABPB1 mRNA.

## Discussion

**Figure 6:**
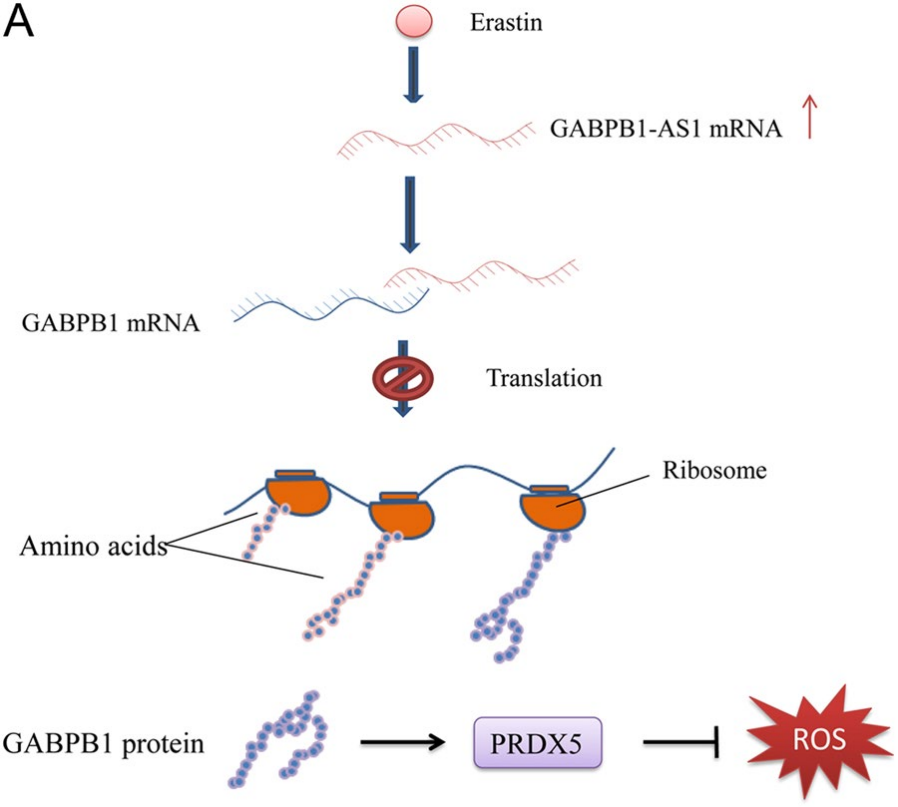
Proposed model of how GABPB1-AS1 regulates GABPB1 translation. (**A**) LncRNA GABPB1-AS1 is upregulated by erastin and forms RNA duplexes with GABPB1 mRNA to then inhibit GABPB1 translation. This results in reduced expression of PRDX5, which ultimately leads to the accumulation of ROS.

Our study demonstrated that erastin upregulated the expression of the GABPB1-AS1 lncRNA and, consequently, inhibited the translation of GABPB1, which resulted in reduced expression of the PRDX5 peroxidase gene and subsequent rapid accumulation of ROS levels in HepG2 cells (Fig. 6A). Overexpression of GABPB1-AS1 had similar effects.

A key process in ferroptosis is the accumulation of hydroxyl radicals produced from hydrogen peroxide by the Fenton reaction, which initiates the lipid peroxidation process and results in membrane lipid damage^48^. Peroxidase is the key metabolic enzyme that controls the excessive accumulation of hydroxyl radicals^49,50^. Downregulation of peroxidase expression directly and negatively impacts the cellular antioxidative ability, which exerts an effect on the antioxidant capacity of hepatocellular carcinoma cells prior to that of reducing equivalent. Our work suggests that the GABPB1-AS1 lncRNA may play an important role in ferroptosis induced by erastin. In further support of the important role of GABPB1 and GABPB1-AS1 in HCC tumourigenesis, our data indicate that high expression levels of GABPB1 are positively correlated with poor prognosis of HCC patients, while high levels of GABPB1-AS1 correlated with improved overall survival.

In addition to the effects of erastin on inhibiting GABPB1 translation by upregulating GABPB1-AS1 lncRNA, we found that erastin could directly inhibit the transcription of GABPB1, leading to decreased mRNA levels. We speculate that GABPB1 may play a role in regulating the interaction between mitochondria and the nucleoplasm and maintains the balance between the energy metabolism of cells and the ability to resist oxidative damage^51^. Erastin interrupts this mechanism and inhibits the cellular response to oxidative stress by downregulating the expression of GABPB1. This effect may be achieved through the role of erastin in VDAC2/3 regulation^51^. We will elucidate this mechanism of erastin in future studies.

## Supplementary information

supplementary information
