# Supplementary material for: LncRNA GABPB1-AS1 and GABPB1 regulate oxidative stress during erastin-induced ferroptosis in HepG2 hepatocellular carcinoma cells
Source: Sci Rep. 2019 Nov 7;9:16185. doi: 10.1038/s41598-019-52837-8 (PMC6838315; doi:10.1038/s41598-019-52837-8)
Supplement: Supplementary file 1 — supplementary information [file 41598_2019_52837_MOESM1_ESM.pdf]

Supplementary Information

Manuscript title

LncRNA GABPB1-AS1 and GABPB1 regulate oxidative stress during erastin-induced ferroptosis in HepG2 hepatocellular carcinoma cells

Wenchuan Qi[1, a], Zhenhua Li[2, a], Longjiang Xia[3], Jiangshan Dai[1], Qiao Zhang[4], Chuanfang Wu[1], Si Xu[1,5*]

[a] These authors contributed equally to the paper.
***Correspondence:** Si Xu (Email: bearpp@126.com)

[1]Key Laboratory of Bio-Resource and Eco-Environment of Ministry of Education, College of Life Sciences, Sichuan University,610065, Chengdu, Sichuan, P.R. China
[2]Changchun University of Traditional Chinese Medicine,130117, Changchun, Jilin, P.R. China
[3]Chengdu University of Traditional Chinese Medicine, 611130, Chengdu, Sichuan, P.R. China
[4]China-Japan Friendship Hospital Affiliated Jilin University, 130033, Changchun, Jilin, P.R. China
[5]Sichuan Academy of Medical Sciences and Sichuan Provincial People's Hospital, 610072, Chengdu, Sichuan, P.R. China

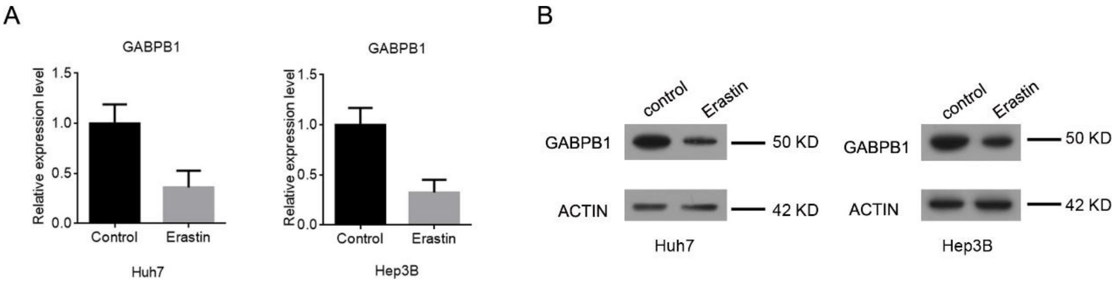

Fig S1. Decreased GABPB1 expression levels during ferroptosis in Huh7 and Hep3B cells. (a, b) Huh7 and Hep3B cells were treated with erastin (10 μM) for 24 h, and GABPB1 mRNA (a) and protein (b) levels were determined. Values are expressed as the means ± SD (n=3). **P* < 0.05, ***P* < 0.01.

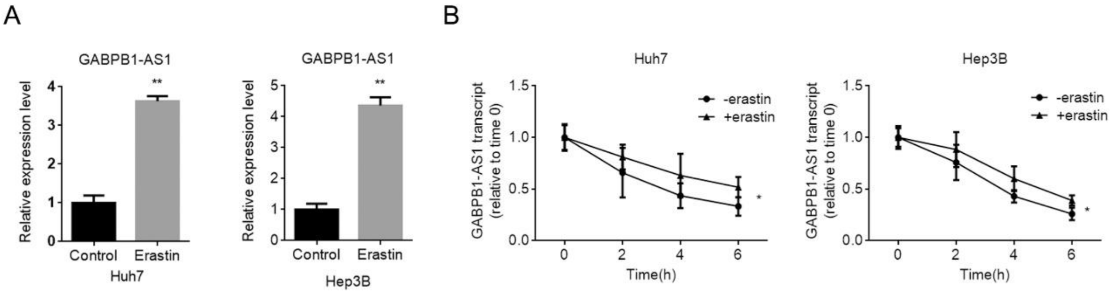

Fig S2. Increased GABPB1-AS1 expression levels during ferroptosis in Huh7 and Hep3B cells. (a) Huh7 and Hep3B cells were treated with erastin (10 μM) for 24 h, and GABPB1-AS1 mRNA levels were determined. (b) The stability of GABPB1-AS1 mRNA over time was measured after blocking new RNA synthesis with α-amanitin (50 μM) and then treated with (+) or without (-) erastin. GABPB1-AS1 mRNA levels were normalized to 18S rRNA levels. Values are expressed as the means ± SD (n=3). **P* < 0.05, ***P* < 0.01.

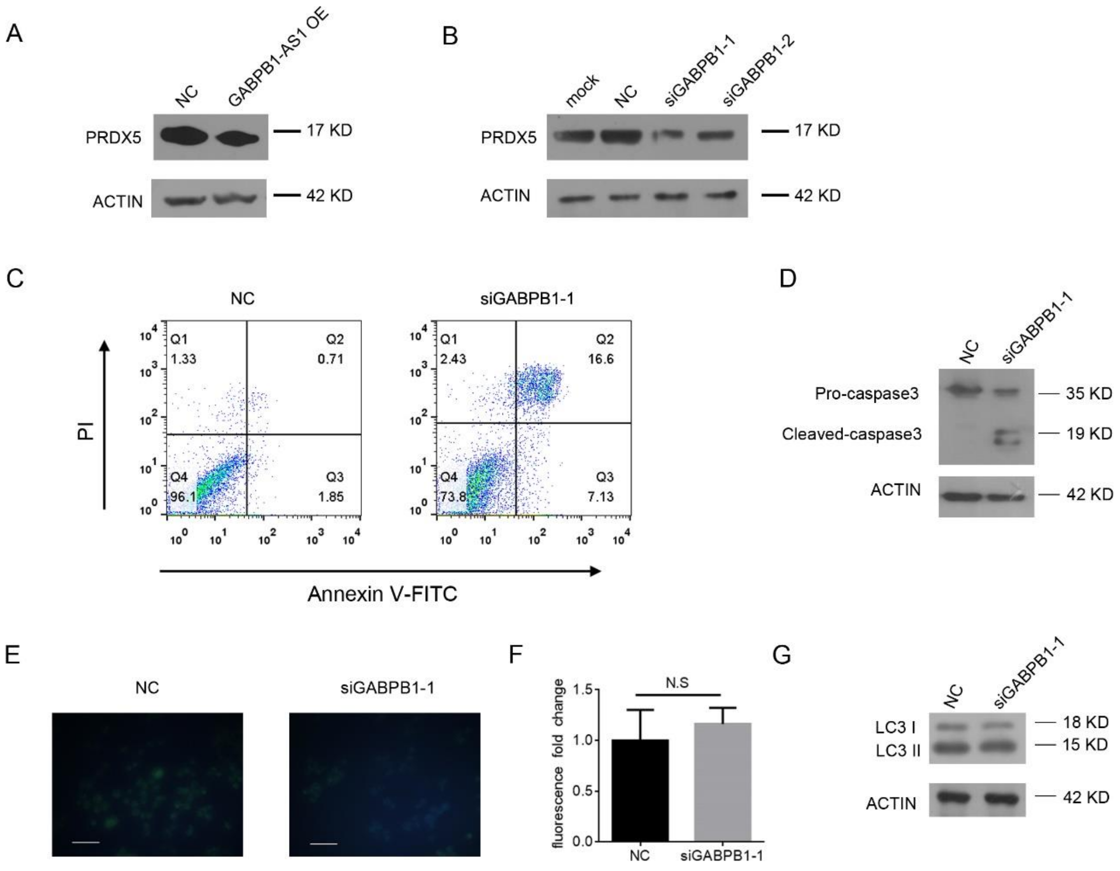

Fig S3. Knockdown of GABPB1 leads to cell apoptosis but not autophagy. (a) Western blot analysis of PRDX5 in HepG2 cells transfected with pcDNA3.1 or GABPB1-AS1 overexpression vector. (b) Western blot confirmed PRDX5 knockdown in HepG2 cells transfected with two different siRNAs for GABPB1. (c) HepG2 cells were transfected with siGABPB1-1, and flow cytometry analysis was performed with Annexin V-FITC/PI staining. (d) Western blot analysis of the apoptosis marker caspase-3 in HepG2 cells. (e) HepG2 cells were transfected with siGABPB1-1, and AVOs (acidic vesicular organelles) were visualized using MDC staining (scale bar: 50 μm). (f) Fold change in MDC fluorescence was measured by a fluorescence microplate reader. (g) LC3 I and LC3 II expression levels were measured using western blot analysis. Values are expressed as the means ± SD (n=3). $^{*}P < 0.05$, $^{**}P < 0.01$.

Supplementary Figure S4 Uncropped images of blots and gels in the article.

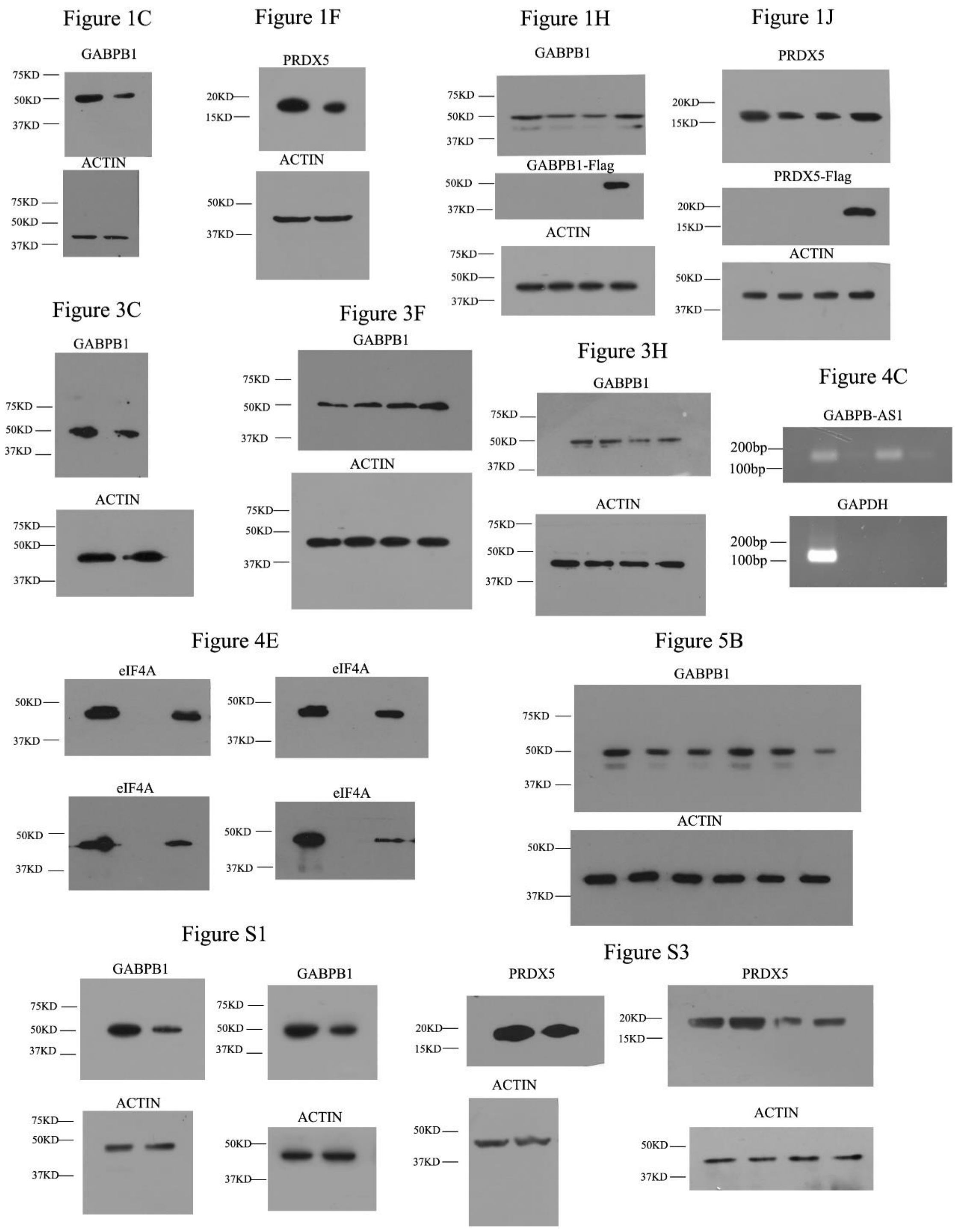

Table 1
Primers and oligonucleotides used in this study.

Name	Sequence (5′-3′)
GABPB1-qPCR-F	TCCACTTCATCTAGCAGCACA
GABPB1-qPCR-R	GTAATGGTGTTCGGTCCACTT
GABPB1-clone-F	CGCGATATCATGTCCCTGGTAGATTTGGGA
GABPB1-clone-R	CCGCTCGAGACTAATAAAGAAGCTGTTTAA
PRDX5-clone-F	CCAAGGATCCAGTGGCCGTGGGGCGGGTATGGG
PRDX5-clone-R	TCACGAATTCTCAGAGCTGTGAGATGATATTGG
PRDX5-ChIP-F	GCACAAACTCTCGCCAGG
PRDX5-ChIP-R	CTGCTGAGACGCTTCCGGTAG
GABPB1-AS1-qPCR-F	GTTCTGCGGTGGGTAGGAGT
GABPB1-AS1-qPCR-R	CCTCTTGGCTGTCCTTCCGTCTC
GABPB1-AS1-clone-F	CTAGCTAGCTGATGAACTGTGCCCTGGCAGAG
GABPB1-AS1-clone-R	CGCGGATCCGAGACGGAGTCTCGCTCTGTCGC
GAPDH-qPCR-F	ATCACCATCTTCCAGGAGCG
GAPDH-qPCR-R	CAAATGAGCCCCAGCCTTC
GABPB1 siRNA1	GCAACACCACAGTTTATCA
GABPB1 siRNA2	GGTGCCATTCAGCAAGTAG
GABPB1-AS1 siRNA1	CUGUUGAAAGACGGAGAGUTT
GABPB1-AS1 siRNA2	AUGGCUUUCCCAACCUAGUTT
negative control (NC) siRNA	UUCUCCGAACGUGUCACGUTT
GABPB1 sense-clone 1stF	GCTTTCTTTGTGTGGCTGAA
GABPB1 sense-clone 1stR	CTGGAAAAGCTTCGGGAGCG
GABPB1 sense-clone 2ndF	TAATACGACTCACTATAGGGGCTTTCTTTGTGTGGCTGAA
GABPB1 sense-clone 2ndR	CTGGAAAAGCTTCGGGAGCG
GABPB1 antisense-clone 1stF	CTGGAAAAGCTTCGGGAGCG
GABPB1 antisense-clone 1stR	GCTTTCTTTGTGTGGCTGAA
GABPB1 antisense-clone 2ndF	TAATACGACTCACTATAGGGCTGGAAAAGCTTCGGGAGC
GABPB1 antisense-clone 2ndR	GCTTTCTTTGTGTGGCTGAA
RPA Overlap region-qPCR-F	GAGAGGGGAAAGAGGGTCTGA
RPA Overlap region-qPCR-R	GCTTTCTTTGTGTGGCTGAAG
RPA Non-overlap region-qPCR-F	GTTCTGCGGTGGGTAGGAGT
RPA Non-overlap region-qPCR-R	CCTCTTGGCTGTCCTTCCGTCTC
